# Transorbital Stab Injury with Retained Knife: A Narrow Escape

**DOI:** 10.1155/2014/754053

**Published:** 2014-09-23

**Authors:** Muhammad Asim Rana, Abdulrehman Alharthy, Waleed Tharwat Aletreby, Basim Huwait, Akhilesh Kulshrestha

**Affiliations:** ^1^Department of Intensive Care Medicine, King Saud Medical City, Riyadh, Saudi Arabia; ^2^Department of Radiology, King Saud Medical City, Riyadh, Saudi Arabia

## Abstract

Transorbital penetrating injuries are unusual but may cause severe brain damage if cranium is entered. These kinds of injuries are dangerous as the walls of orbit are very thin, hence easily broken by the otherwise innocent objects. Because of the very critical anatomical area involved, these injuries pose a serious challenge to the physicians who first receive them as well as the treating team. These may present as trivial trauma or may be occult and are often associated with serious complications and delayed sequel. Prompt evaluation by utilizing best diagnostic modality available and timely interference to remove them are the key aspects to avoid damage to vital organs surrounding the injury and to minimize the late complications. We report a case of transorbital assault with a 13 centimeter long knife which got broken from the handle and the blade was retained. The interesting aspect is that there was no neurological deficit on presentation or after removal.

## 1. Case Report

A 59-year-old male was shifted from another hospital via ambulance after being stabbed in his left eye.

**Figure 1:**
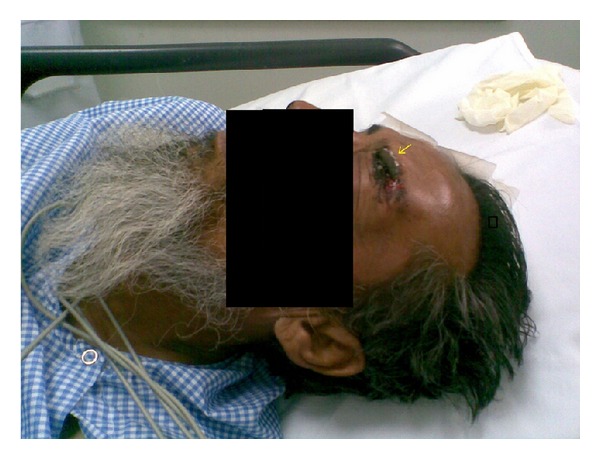
Figure showing the broken end of knife (yellow arrow).

The patient when received was fully conscious, oriented, and hemodynamically stable. A gross pen torch examination revealed a retained knife broken from the handle entering the supraorbital region of the left eye near the medial canthus. Only one and a half centimeters of handle was visible outside (Figure 1). There was complete ptosis with some ecchymosis of the upper eye lid. The eye ball was intact with no signs of corneal or anterior scleral perforation. Other physical, neurological, and ophthalmological examinations revealed no abnormality.

**Figure 2:**
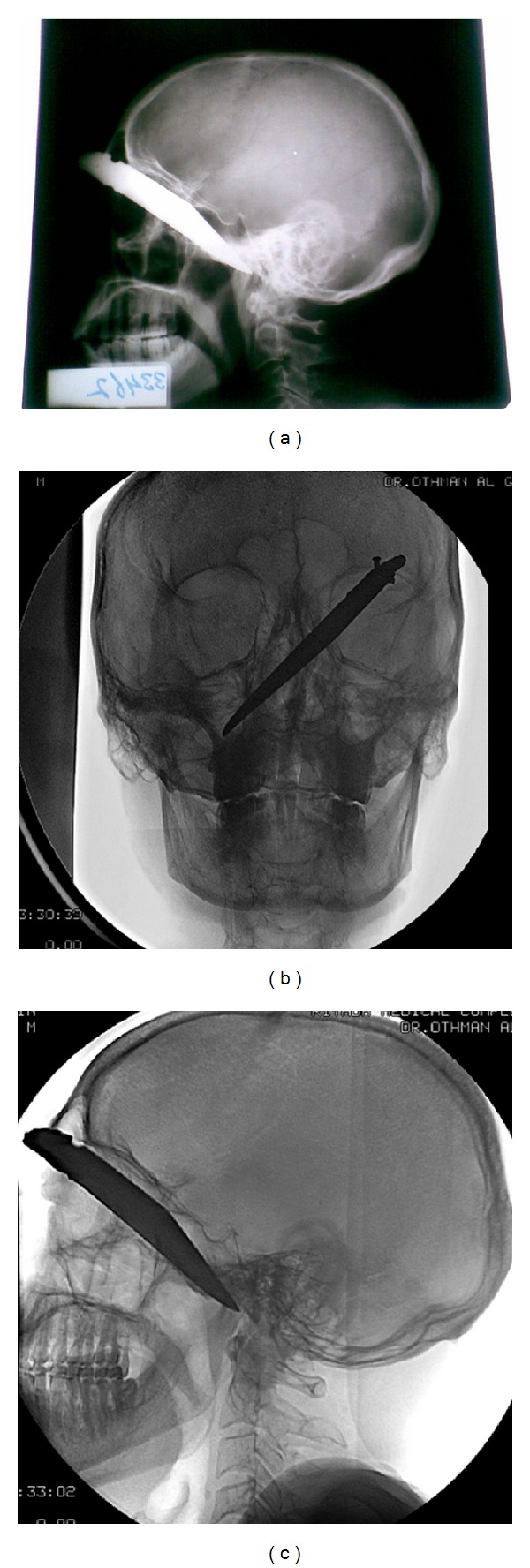
((a), (b), and (c)) Radiographic images describing the route of the knife and the tip reaching the infratemporal region across the midline.

Initial images showed the tip of the knife crossing the midline and reaching the infratemporal region on right side (Figures 2(a), 2(b), and 2(c)).

CT scan was temporarily unavailable because of some technical issues so an urgent four-vessel angiogram was carried out to see if any vascular involvement was there.

**Figure 3:**
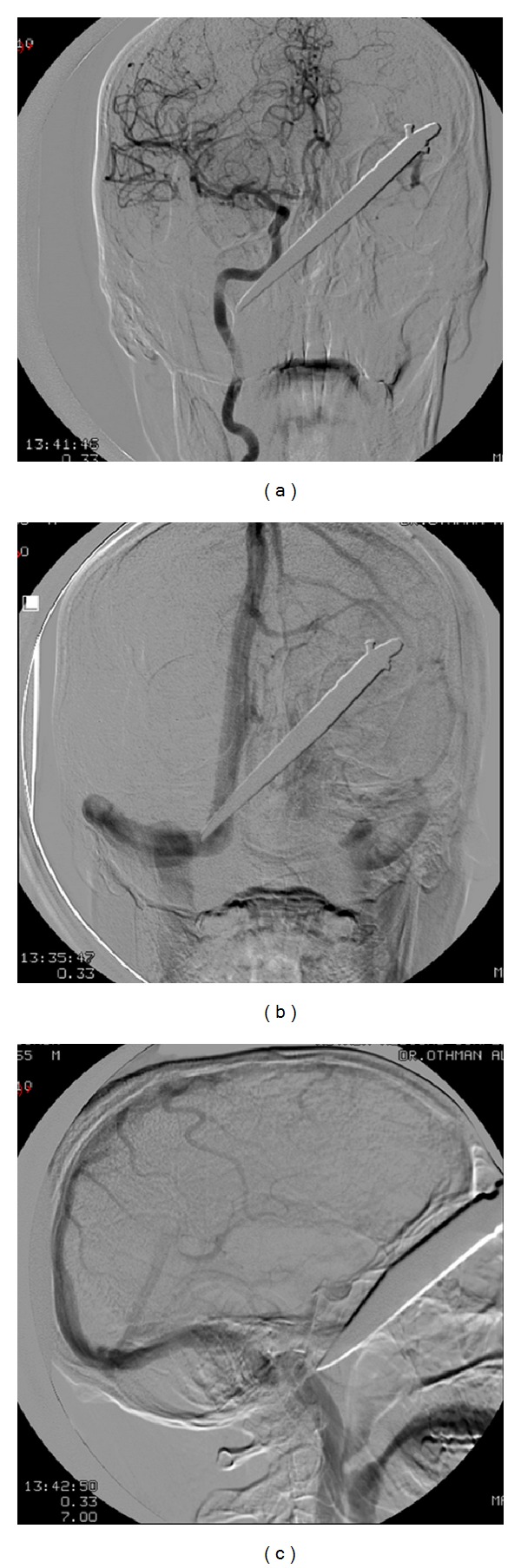
((a), (b), and (c)) Angiogram to evaluate the integrity of vasculature. The tip of knife is visible near left internal carotid artery. The venous sinus and internal jugular vein appear safe.

The four-vessel angiogram showed a big knife entering the facial soft tissue from left supraorbital region extending posteriorly and downwards across the face and the tip ending at the infratemporal region on right side. All vessels, including right internal and external carotid arteries, left internal and external carotid arteries and vertebrobasilar system were found to be intact. Venous sinuses and cervical veins were also normal. No contrast leakage or aneurysm was found (Figures 3(a), 3(b), and 3(c)).

The patient was promptly taken to operation theatre and the knife was removed by a combined surgical operation carried out by maxillofacial and neurosurgery teams.

For removal of knife, bifrontal craniotomy was done and left superior orbital rim was removed. The knife was found stuck deep in the bony part of medial orbital rim which was removed to loosen the knife blade. Soft tissues around the blade were dissected and maximum possible length was exposed. Knife was then pulled with gentle force. No bleeding was seen. Area was irrigated with saline and the medial and superior orbital rims were fixed back using screws and plates.

Postoperative recovery was uneventful and there were no neuroophthalmological deficits at long term follow-up.

## 2. Discussion

Penetrating head and neck trauma especially when transorbital causes uncommon but potentially life threatening injuries and it is a challenging situation both for the initially evaluating physician as well as for the surgeon as a prompt evaluation and management of such a condition are essential to preserve vital visual functions and save life.

Penetrating head and neck injuries have been classified for long into high velocity and low velocity injuries.

High velocity injuries usually are acquired in war caused by missile injuries like gun shots and shrapnel wounds and they cause massive destruction of the cranium and face.

Most of civilian injuries are low velocity and are caused by otherwise innocent objects. There have been multiple reports of such objects like toys, pencils, stones, wooden sticks, bicycle brake handle, chopsticks, umbrella ends, thumb tacks, tooth brushes, crochet hooks, and metal fence [1–9], while on the other hand reports on direct transorbital stab injuries by knife are few [10–14].

The risk of these kinds of injuries is high especially in the orbital region because the orbital roof and medial wall are relatively thin and the objects even with less force can easily penetrate deep and cause damage to the globe, brain, cavernous sinus, paranasal sinuses, and optic nerve. Hence, the pathophysiological consequences and degree of permanent neurological deficit in such injuries depend upon the kinetic energy and the pathway or trajectory of the offending object, timing to access the medical care, rapidity of exploration, removal of the object and avoiding the secondary injury [15, 16]. The consequences of such wounds include brain contusions, cerebrospinal fluid fistulas, intracerebral, subdural, and extradural hematomas, and pneumocephalus. Late sequel can be infectious complications like encephalitis, meningitis, or cerebral abscess [17–21]. Vascular malformations although rare can ensue [22, 23]. The outcome of such injuries is also dependent on primary injury and its associated complications. Subarachnoid haemorrhage for instance has been associated with poor outcome [24].

The diagnosis of such injuries is straight forward if the precise knowledge of the traumatic event and the nature of the object are available or presence of the foreign body can be confirmed in the wound. However, diagnosis based on an incomplete history and in cases of trivial trauma is difficult and the penetrating injuries may be overlooked [25–27].

In our case, we were able to obtain a detailed history of the event and the object was visualized in situ. Although the clinical examination including both ophthalmological and neurological components is crucial in determining the site and effect of the injury, imaging techniques are extremely helpful in making final decision and embarking upon a method of removal. Computerized tomography is an excellent means of documenting details of orbitocranial trauma such as extent of soft tissue damage, localization and nature of the foreign material, and presence of bone fractures and is indicated in all cases of suspected cranial penetration.

In our case, we could not perform a CT scan because of a temporary technical issue but we did a four-vessel angiogram to rule out any vascular involvement.

As far as the removal of the objects retained in the orbit with possible cerebral penetration is concerned, the procedures described in the literature range from simple extraction to surgical removal depending upon the size, nature, and location of the object because the considerable differences in the shape and size of the different objects involved in such accidents make it impossible to establish a single therapeutic strategy. Some authors have the opinion that if the object is small and the shape is known, extraction can be attempted, while surgical removal is advised in other cases [12, 21, 28–32].

As the wound of entry was high enough through the supraorbital area, the surgery was attended by the oromaxillofacial and neurosurgery teams and it proved to be the best way as they had to do craniotomy and open the orbital rim for the removal of the knife blade.

In the case, we reported the traumatic cranial damage was exclusively of primary type and the intracranial extension of injury which is usually a common occurring in such violent and dramatic traumas did not take place. It was indeed a narrow escape.

The case should heighten the awareness of first responders to such conditions about the possibilities of vascular injuries or brain stem damage as the inner extent of the foreign object is not known and they should arrange safe transfer of such patients to the centers where the treatment facilities are available. Moreover, diagnostic tools should be appropriately used and a multidisciplinary team approach should be sought for the best management.
